# 130例青年肺癌的临床病理特征

**DOI:** 10.3779/j.issn.1009-3419.2014.06.05

**Published:** 2014-06-20

**Authors:** 姝宁 宫, 崇铃 桑, 志勇 徐, 玉红 王

**Affiliations:** 037006 大同，人民解放军322医院呼吸内科 Department of Respiratory Medicine, No. 322 Hospital of the People's Liberation Army, Datong 037006, China

**Keywords:** 肺肿瘤, 青年人, 临床, 病理, Lung neoplasms, Young patient, Clinical, Pathological

## Abstract

**背景与目的:**

青年肺癌病情进展快，及时准确的诊断十分重要。本研究旨在通过探讨青年肺癌的临床和病理特征，为及时诊断提供线索。

**方法:**

研究纳入1995年-2012年在人民解放军322医院经病理学和细胞学诊断的年龄 < 40岁的肺癌患者，回顾性分析其性别、年龄、症状、吸烟史、病理类型及分化程度、临床分期及误诊情况。

**结果:**

本组共130例，占同期所有肺癌的5.2%。其中男性68.5%；36岁-40岁占53.8%。就诊时的症状以咳嗽、痰中带血等局部症状最为常见，非特异性全身表现相对少见；无症状患者约占11.8%。有吸烟史者占63.3%。从出现症状到经病理学或细胞学确诊平均经历3.9个月，其中51.5%在最终确诊前发生过误诊，最常见的误诊为结核。Ⅲ期/Ⅳ期患者占85.4%。原发病灶以右肺上叶和左肺上叶多见；腺癌最为常见；低分化癌占72.3%。

**结论:**

青年肺癌的临床病理特征具有一定特殊性。对于拟诊为结核的患者，尤其是全身症状不明显的患者，应考虑到肺癌的可能性并行病理学和细胞学检查。无吸烟史不能排除青年肺癌的可能性。

## Abstract (en)

**Background and objective:**

It has been proven that lung cancer in the youth exhibited rapid progression, thus timely and accurate diagnosis is crucial. The aim of this study is to explore the clinicopathological characteristics of lung cancer in the youth, so as to provide clues for diagnosis.

**Methods:**

The study enrolled patients aged younger than 40 years with lung cancer diagnosed with histology or cytology in our hospital between 1995-2012. We retrospectively analyzed sex, age, symptoms, smoking history, histology, stage and misdiagnosis.

**Results:**

One hundred and thirty patients with lung cancer in the youth were enrolled, which comprise 5.2% of all cases of lung cancer in that period. Among them, 68.5% were male; 53.8% were aged between 36 and 40 years. Common symptoms included cough and hemoptysis, and systematic symptoms were relatively uncommon; and 11.8% showed no symptoms. 63.3% had a smoking history, and the time between initial symptom and final diagnosis averaged 3.9 months. The misdiagnosis rate was 51.5%, with tuberculosis as the leading misdiagnosis. 85.4% were staged Ⅲ/Ⅳ. Primary lesion often located in upper lobes. Adenocarcinoma was the commonest histology, and 72.3% of the whole group was poorly differentiated.

**Conclusion:**

Lung cancer in the youth showed some special clinicopathological characteristics. Doctors should consider the possibility of lung cancer in patients with seemly diagnosis of tuberculosis, especially in those without symptomatic symptoms, and perform histological or cytological examinations. The absence of smoking history cannot rule out the possibility of lung cancer in youth patients.

近年来肺癌在我国的发病率和死亡率急剧上升，已经位居我国肿瘤发病率和死亡率的首位^[1]^。我们在临床实践中发现肺癌的发病有年轻化的趋势。青年肺癌病情进展快，及时准确地诊断将为患者争取宝贵的治疗时机。但实际上青年肺癌的误诊率较高，重要原因之一是此病并非青年人群的常见疾病，对其临床和病理特征研究有限，从而影响了与其他疾病的鉴别。本研究总结单中心130例青年肺癌的临床和病理学特征，以期为及时诊断提供线索。

## 1 资料与方法

### 1.1 患者与资料

研究纳入1995年-2012年经人民解放军322医院诊断的年龄 < 40岁的肺癌患者，所有患者均经病理学或细胞学检查确诊，并且临床资料完整。

收集所有患者的以下数据：性别、诊断肺癌时的年龄、症状及其持续时间、就诊经历、吸烟史、病理类型及分化程度、临床分期、是否存在远处转移及转移部位。其中吸烟史统计有无吸烟史，以及吸烟指数（定义为每天吸烟包数乘以吸烟年数）。症状持续时间定义为自症状首次出现到获得病理学或细胞学诊断的时间。分期标准采用国际抗癌联盟（International Union Against Cancer, UICC）的TNM分期（第7版）^[2]^。

### 1.2 统计学分析

采用SPSS 11.5软件包进行统计。各发生率的比较使用χ^2^检验，*P* < 0.05为差异有统计学意义。

## 2 结果

### 2.1 青年肺癌在所有肺癌中的构成比和发病变化趋势

自1995年-2012年共经病理学或细胞学确诊肺癌2, 507例，其中130例肺癌诊断时年龄 < 40岁，占5.2%。1995年-2002年诊断青年肺癌49例，而2003年-2012年诊断青年肺癌81例，较1995年-2002年增长了65.3%；其中女性增长41.2%（1995年-2002年17例，2003年-2012年24例），男性增长87.1%（1995年-2002年31例，2003年-2012年58例）；各时间段中均以男性病例为多；组织病理类型鳞状上皮细胞癌（以下简称鳞癌）降低7.7%（1995年-2002年13例，2003年-2012年12例），腺癌增长57.6%（1995年-2002年33例，2003年-2012年52例），小细胞癌增长175.0%（1995年-2002年4例，2003年-2012年11例），其他类型1995年-2002年1例，2003年-2012年4例）。

### 2.2 青年肺癌的临床特征

#### 2.2.1 一般情况

本组中男性89例（68.5%），女性41例（31.5%），男女比例为2.17:1。诊断年龄平均为34.3岁，最小者仅为14岁。病例在各年龄段的分布为：14岁-20岁3例（2.3%），21岁-25岁5例（3.8%），26岁-30岁23例（17.7%），31岁-35岁29例（22.3%），36岁-40岁70例（53.8%）。

#### 2.2.2 临床症状

患者就诊时的症状以咳嗽、痰中带血等局部症状最为常见，部分患者出现远处转移症状和非特异性全身表现（如发热和盗汗），少数患者并无症状。其中咳嗽68例（52.3%），痰中带血31例（23.9%），胸痛29例（22.3%），胸闷16例（12.3%），发热11例（8.4%），气促8例（6.2%），骨痛7例（5.4%），头痛6例（4.6%），声嘶3例（2.3%），吞咽困难1例（0.8%），盗汗1例（0.8%），无症状14例（10.8%）。

#### 2.2.3 吸烟史

本组中有吸烟史者57例（63.3%），其中男性53例，女性4例；吸烟指数 > 20包年者31例，吸烟指数 > 10包年者19例。

#### 2.2.4 诊断和误诊

从出现症状到经病理学或细胞学确诊所经历的时间，最短为1周，最长为59个月，平均3.9个月。其中67例（51.5%）在最终确诊前发生过误诊，分别为结核性胸膜炎（23例）、肺结核（27例）、肺炎（11例）、肋间神经痛（3例）和关节炎（3例）。

#### 2.2.5 临床分期和转移部位

病例分期如下：Ⅰ期9例（6.9%），Ⅱ期10例（7.7%），Ⅲ期54例（41.5%），Ⅳ期57例（43.9%）。有72例发生远处转移，其中骨转移34例（26.2%）、肺转移14例（10.8%）、脑转移12例（9.2%）、肝转移9例（6.9%）、肾上腺转移2例（1.5%）、眼转移1例（0.8%）。

### 2.3 青年肺癌的病理特征

#### 2.3.1 原发病灶部位

本组青年肺癌发生于右肺81例（62.3%），左肺49例（37.7%），两者间差异有统计学意义（*P* < 0.05）；以右肺上叶和左肺上叶多见，分别为42例和29例。

#### 2.3.2 组织学类型及分化程度

本组中腺癌最为常见，其次为鳞癌，小细胞癌居第3位；低分化癌（包括低分化癌、腺癌、小细胞癌和大细胞癌）94例（占72.3%），高分化癌17例（13.1%），两者差异有统计学意义（*P* < 0.01）（表 1）。本组中各病理类型均为男性多于女性，男女发病率比分别为：小细胞癌为3.2:1，鳞癌为2.8:1，腺癌为1.7:1。发生转移的病例中腺癌占45例（62.5%），鳞癌7例（9.7%），小细胞癌5例（6.9%），大细胞癌2例（2.8%），类癌1例（1.4%）。

**1:** 青年肺癌的组织学类型 Histology of lung cancer in the youth

Histology	Number	Percentage
Squamous	22	16.9%
Well differentiated	5	3.9%
Moderately differentiated	2	1.5%
Poorly differentiated	15	11.5%
Adenocarcinoma	84	64.6%
Well differentiated	12	9.2%
Moderately differentiated	8	6.2%
Poorly differentiated	64	49.2%
Small cell	13	10.0%
Large cell	2	1.5%
Carcinoid	3	2.3%
Bronchial gland carcinoma	4	3.1%
Adenosquamous	2	1.5%

## 3 讨论

青年肺癌在所有肺癌中仅占少数，但患者正处于能为社会做出贡献的年龄段，并且在我国独生子女政策的背景下，会对整个家庭造成严重影响。国内外文献^[3, 4]^表明，青年肺癌存在着特殊的临床病理特征，但各研究的结论并不完全一致，这可能是由于研究病例数较少，各研究中可手术患者的比例不同，以及各地的遗传、种族、吸烟、环境暴露因素存在差异。文献^[5-9]^报道青年肺癌占所有肺癌的1.2%-10.0%，本组中40岁以下肺癌占同期肺癌总数的5.2%，与文献相符。但值得注意的是，由于我国吸烟持续得不到控制，空气污染日趋严重，这一数字可能将上升。

本组中所有患者均为40岁以下，随年龄增长，发生肺癌的例数递增，提示对于年龄较长的青年，应更加重视发生肺癌的可能性。患者中以男性多见，这可能是由于男性吸烟比例高，并且更易接触职业污染。欧美地区青年肺癌患者中85%-97%有吸烟史^[10, 11]^，而在亚洲此比例仅为41%-66%^[5, 12]^。本研究中吸烟比例印证了既往亚洲地区报告的结果。这提示非吸烟因素在我国青年肺癌的发病中也发挥重要作用；值得强调的是，对于无吸烟史的青年患者，并不能排除肺癌的诊断。

本组患者的临床表现以咳嗽、痰中带血、胸痛最为常见，与既往文献^[6]^报告一致。近半数的患者在诊断为肺癌前发生过误诊。最多见的误诊是结核，这也与我国其他文献^[13]^报告一致。此现象可能与结核在青年患者中更常见有关。并且，青年肺癌的好发部位是上肺，结核亦常见于上肺，是造成混淆的原因之一。因此，对于青年患者发生于上肺的病变，不能贸然诊断为结核，而应进行详细地检查，考虑到发生肺癌的可能性。值得注意的是，青年肺癌患者的全身症状如发热、盗汗并不明显，这与结核存在明显差异，可以作为鉴别诊断的参考。本研究表明，有约10%的青年肺癌患者，并无症状而是由常规体检发现，提示青年患者中常规体检的重要性。确诊肺癌决定性的检查仍是病理学或细胞学检查。

本组低分化癌约占70%，以腺癌和小细胞癌转移为主，与既往文献^[14, 15]^相符。肿瘤分化程度低，意味着生长迅速，若未经有效治疗预后不佳。另一方面，青年肺癌患者合并症少，对治疗的耐受性较好，积极治疗有望改善预后。对于可手术患者，应积极给予手术治疗。对于晚期患者，在治疗靶点检测的基础上给予个体化治疗已经成为大势所趋。对于青年肺癌发病机制和治疗靶点的研究将为阐明此病的特质和发现更有效的治疗手段提供线索。有趣的是，近期的一项小宗病例研究^[16]^表明青年肺癌患者中驱动基因的阳性率似乎较高。

综上，青年肺癌的发病风险随年龄增长而递增，以男性多见，腺癌是最常见的病理类型，以低分化癌为主。青年肺癌常易误诊，医务人员应提高对青年肺癌的认识，尤其是对拟诊为结核的患者，应审慎考虑临床表现是否有难以解释之处，并进行有针对性的辅助检查，包括病理检查和细胞学检查，以及早诊断青年肺癌并给予合适的治疗，最终改善患者的预后。
